# Nano-titanium dioxide modulates the dermal sensitization potency of DNCB

**DOI:** 10.1186/1743-8977-9-15

**Published:** 2012-05-23

**Authors:** Salik Hussain, Stijn Smulders, Vanessa De Vooght, Bert Ectors, Sonja Boland, Francelyne Marano, Kirsten L Van Landuyt, Benoit Nemery, Peter HM Hoet, Jeroen AJ Vanoirbeek

**Affiliations:** 1 Unit of Functional and Adaptive Biology (BFA) CNRS EAC 4413, Laboratory of Molecular and Cellular Responses to Xenobiotics (RMCX), Univ Paris Diderot, Sorbonne Paris Cité, France; 2 Department of Public Health, Occupational, Environmental and Insurance Medicine, KU Leuven, Herestraat 49 bus 706, Leuven 3000, Belgium; 3 KU Leuven BIOMAT, Department of Oral Health Sciences, Conservative Dentistry, KU Leuven, Leuven, Belgium; 4 Present Address: Clinical research unit, National Institute of Environmental Health Sciences (NIEHS)/National Institute of Health (NIH), Research Triangle Park, North Carolina, USA

**Keywords:** Nanoparticle, Titanium dioxide (TiO_2_), Lymph node proliferation assay (LNPA), DNCB, Skin sensitization

## Abstract

We determined the ability of a model nanoparticle (NP) (titanium dioxide, TiO_2_) to modulate sensitization induced by a known potent dermal sensitizer (dinitrochlorobenzene) using a variant of the local lymph node assay called lymph node proliferation assay.

BALB/c mice received sub-cutaneous injections of vehicle (2.5 mM sodium citrate), TiO_2_ NPs (0.004, 0.04 or 0.4 mg/ml) or pigment particles (0.04 mg/ml) both stabilized in sodium citrate buffer at the base of each ear (2x50μl), before receiving dermal applications (on both ears) of 2,4-Dinitrochlorobenzene (DNCB) (2x25μl of 0.1%) or its vehicle (acetone olive oil – AOO (4:1)) on days 0, 1 and 2. On day 5, the stimulation index (SI) was calculated as a ratio of ^3^HTdR incorporation in lymphocytes from DNBC-treated mice and AOO-treated controls. In a second experiment the EC_3_-value for DNCB (0 to 0.1%) was assessed in the absence or presence of 0.04 mg/ml TiO_2_. In a third experiment, the lymphocyte subpopulations and the cytokine secretion profile were analyzed after TiO_2_ (0.04 mg/ml) and DNCB (0.1%) treatment.

Injection of NPs in AOO-treated control mice did not have any effect on lymph node (LN) proliferation. DNCB sensitization resulted in LN proliferation, which was further increased by injection of TiO_2_ NPs before DNCB sensitization. The EC_3_ of DNCB, with prior injection of vehicle control was 0.041%, while injection with TiO_2_ decreased the EC_3_ of DNCB to 0.015%. TiO_2_ NPs pre-treatment did not alter the lymphocyte subpopulations, but significantly increased the level of IL-4 and decreased IL-10 production in DNCB treated animals.

In conclusion, our study demonstrates that administration of nano-TiO_2_ increases the dermal sensitization potency of DNCB, by augmenting a Th_2_ response, showing the immunomodulatory abilities of NPs.

## Background

Nanoparticles of titanium dioxide (TiO_2_) are one of the most abundantly produced and widely utilized nanomaterials [1], with applications in sunscreens, cosmetics, tooth pastes, and food products [2-4]. Other important industrial applications include water clean-up technology, oxygen sensor and antimicrobial coatings and ceramics. Titanium nanomaterials have proved their potentials in the fields of drug and gene delivery [2]. Although considered as an inert material, titanium alloys and implants have been shown to release both micrometric and nanometric particles and debris in the surrounding body fluids and tissues which can cause health effects either at the implant site or in distant organs [5-8]. A variety of newly developed house hold products (including self-cleaning spray and paint) have been reported to contain TiO_2_NPs. According to the Project on Emerging Nanotechnologies (Woodrow Wilson International Center for Scholars), as of 10^th^ of March 2011, 1317 consumer products containing NPs are already on the market and if the same trend persists this number is expected to reach 3400 by the year 2020. TiO_2_ is the 3^rd^ largest material used in the consumer products.

Effects of NPs on biological systems are unknown or under debate [9,10]. Nevertheless, the widespread uses of TiO_2_ NPs confirm the possibilities of exposure through ingestion, inhalation and dermal routes. Taken together with the substantial increase of products (in particular skin care products) containing TiO_2_ nanomaterials, there is an urgent need for assessing these newly developed materials for their possible skin sensitizing potentials as well as for their impact on the skin sensitization caused by the other chemicals.

Allergic skin sensitization caused by chemicals is a serious environmental and occupational health hazard. It is the most frequent manifestation of immunotoxicity in humans [11]. Literature reports identify more than 3700 chemical as skin sensitizers [12]. Based on positive skin sensitization tests in animals, it is predicted that in the near future an increase in the number of chemicals capable of causing contact dermatitis in humans will follow.

Dinitrochlorobenzene (DNCB) is a well-known skin sensitizer. It is the most used chemical to study contact hypersensitivity of the skin and the pathogenesis of contact dermatitis [13,14]. Contact dermatitis observed after DNCB application is a T-cell mediated immune response [15]. DNCB formed covalent complexes with various proteins after topical application and act as immunogen which are taken up by antigen presenting cells (APC), processed and presented to T cells for activation.

The Local Lymph Node Assay (LLNA) is an OECD approved protocol to assess dermal sensitizing capacity of chemicals (OECD guideline no. 429, 2002) [16]. A modified version of this test, lymph node proliferation assay (LNPA), has recently been suggested as more appropriate test methodology for the evaluation of NP induced delayed type hypersensitivity reaction [17,18]. The major advantage of LNPA/LLNA includes the possibility to calculate chemicals relative potency of inducing dermal sensitization.

The aim of this study was to evaluate the effect of a prior administration of TiO_2_ NPs on the dermal sensitization potential of DNCB. We hypothesized that TiO_2_ NPs might act as an adjuvant for a skin sensitizer, such as DNCB. To our knowledge, this is the first study to describe the effect of NPs on the dermal sensitization potential of a well-known chemical sensitizer.

## Results

**Figure 1:**
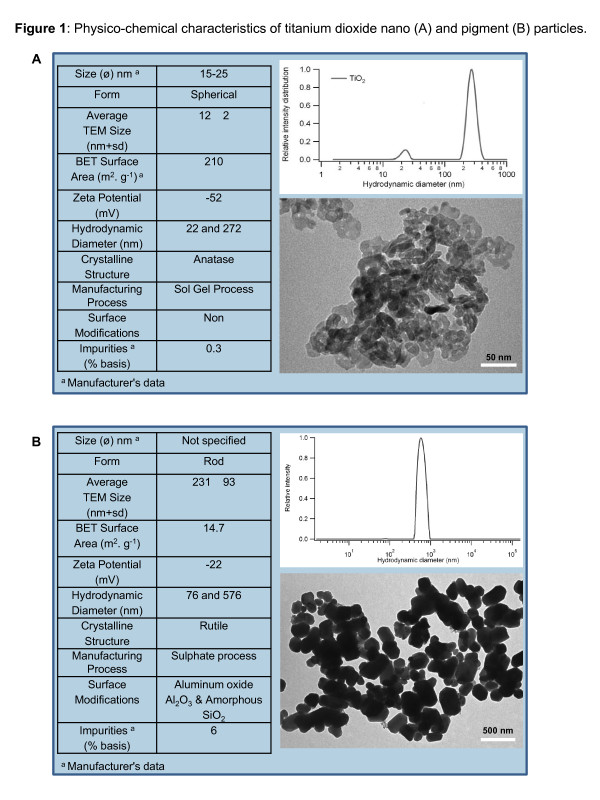
**Physico-chemical characteristics of TiO**_**2**_**nano (A) and pigment (B) particles in powder and suspension.**

Figure 1 shows the details on the particle characteristics. Analysis of homogeneous suspensions of the NPs in 2.5 mM sodium citrate by dynamic light scattering (DLS) showed two populations in the TiO_2_ samples. Primary TiO_2_ particles with a hydrodynamic diameter of 22 nm were detected next to aggregates with a mean hydrodynamic diameter of 272 nm (Figure 1A). Since larger particles scatter more photons than small particles, the intensity weighted distribution is favoured towards larger scatterers. On a number basis, <0.01% of the particles were agglomerates or aggregates. On a mass basis, 23.8% of the mass was in agglomerates or aggregates. It shows that a significant number of TiO_2_ particles exist as primary particles in the suspension. The *ζ* potential for TiO_2_ NPs in 2.5 mM sodium citrate (−52 mV) was significantly lower as compared to *ζ* potential in water (−24 mV) (Figure 1A, inset). This lowered *ζ* potential as compared to that observed in water showed the stabilising effect of sodium citrate on NP suspensions. TEM analysis revealed a spherical morphology of TiO_2_ NPs (Figure 1A, inset). For the pigment TiO_2_ particles, the DLS analysis also revealed two populations. A first population has an average diameter of 576 nm, the second population has an average diameter of 76 nm (Figure 1B). The *ζ* potential for the pigment TiO_2_ particles in 2.5 mM sodium citrate was −22 mV (Figure 1B, inset). The pigment particles also showed a rod-like morphology on TEM analysis (Figure 1B, inset).

**Figure 2:**
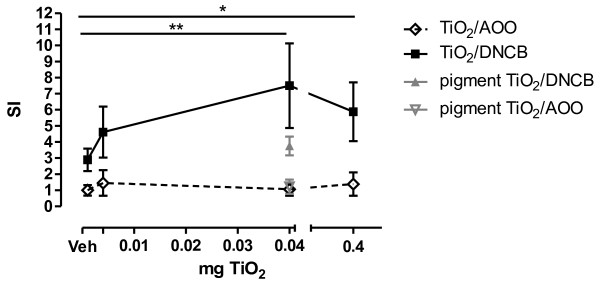
**Effect of subcutaneous injections of TiO**_**2**_**NPs or pigment particles on the skin sensitizing capacity of 0.1% DNCB.** Stimulation Index in lymph node proliferation assay (LNPA). To calculate the SI, all SI’s of the AOO-treated were pooled, since they were not statistically different from each other. Graph shows mean ± SD, n = 5–9, * *p* < 0.05 and ** *p* < 0.01.

Figure 2 shows the modulation effect of different doses of TiO_2_ NPs on the dermal sensitization with DNCB. In AOO-treated control mice, a prior injection with TiO_2_ NP did not influence the SI. When TiO_2_ NPs were injected prior to DNCB sensitization, we found an increasing SI, compared to Veh injection prior to DNCB sensitization. This increased SI was statistically different using 0.04 and 0.4 mg/ml TiO_2_ NPs prior to the DNCB sensitization. Injection of 0.04 mg/ml pigment TiO_2_ particles prior to DNCB sensitization was not significantly different from the group which received an injection with vehicle prior to DNCB sensitization. However, TiO_2_ pigment (0.04 mg/ml) injection prior to DNCB sensitization is statistically different from TiO_2_ NP (0.04 mg/ml) injection prior to DNCB sensitization (*p* < 0.05, not indicated in figure).

**Figure 3:**
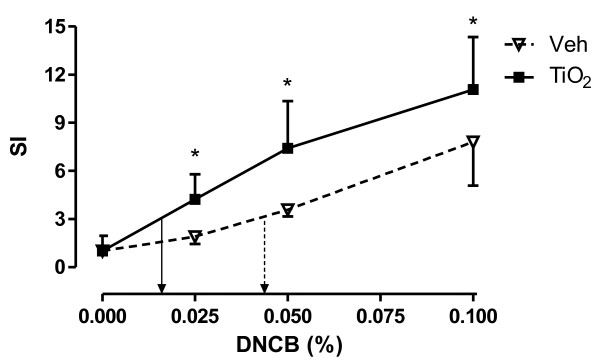
**Effect of subcutaneous injections of 0.04 mg/mlTiO**_**2**_**NPs on the dose–response curve (SI) of DNCB sensitization.** Mice were exposed to different doses of DNCB in the presence or absence of TiO2 NP pre-treatment (0.04 mg/ml). The EC_3_ value of Veh/DNCB is 0.041% (⇢), while the EC_3_ of TiO2/DNCB is 0.015% (→). Graph shows mean ± SD, n = 5-9, *p < 0.05.

Figure 3 shows the effect of prior TiO_2_ NP injection on the EC_3_ value of DNCB sensitization. Prior injection with vehicle (Veh), before sensitization with DNCB yielded an EC_3_ of 0.041%, while injection with TiO_2_ NPs (0.04 mg/ml i.e. 160 μg/kg) before DNCB sensitization led to a shift to the left and resulted in an EC_3_ of 0.015%.

In Table 1, the lymphocyte subpopulations of the auricular lymph nodes are presented. Mice subcutaneously injected with vehicle, followed by 0.1% DNCB sensitization showed significantly increased levels of CD3^+^ (T-cells), CD3^+^CD4^+^ (T-helper cells), CD3^+^CD4^+^CD25^+^ (activated/regulatory T-cells), CD3^+^CD8^+^ (T-cytotoxic cells) and CD19^+^ (B-cells) in the auricular lymph nodes. Prior injection of 0.04 mg/ml TiO_2_ NP before DNCB sensitization did not change the composition of the auricular lymph nodes compared to Veh/DNCB group.

**Table 1:** **Lymph node cell subpopulations (x10**^**6**^**) per lymph node**

	**Veh/AOO**	**Veh/DNCB**	**TiO**_**2**_**/AOO**	**TiO**_**2**_**/DNCB**
**CD3**^**+**^	0.66 ± 0.21	2.06 ± 0.56***	1.16 ± 0.57	2.27 ± 0.59^+++^
**CD3**^**+**^**CD4**^**+**^	0.47 ± 0.15	1.47 ± 0.41***	0.81 ± 0.38	1.56 ± 0.43^+++^
**CD3**^**+**^**CD4**^**+**^**CD25**^**+**^	0.04 ± 0.01	0.16 ± 0.05***	0.06 ± 0.03	0.13 ± 0.02^++^
**CD3**^**+**^**CD8**^**+**^	0.18 ± 0.06	0.59 ± 0.15***	0.34 ± 0.20	0.68 ± 0.19^+++^
**CD19**^**+**^	0.12 ± 0.04	0.62 ± 0.22***	0.23 ± 0.13	0.80 ± 0.30^+++^

Results are expressed as mean ± SD, n = 9-11, ***p < 0.001 Veh/DNCB vs Veh/AOOand ^++^*p* < 0.01, ^+++^*p* < 0.001 TiO_2_/DNCB vs. TiO_2_/AOO.

**Figure 4:**
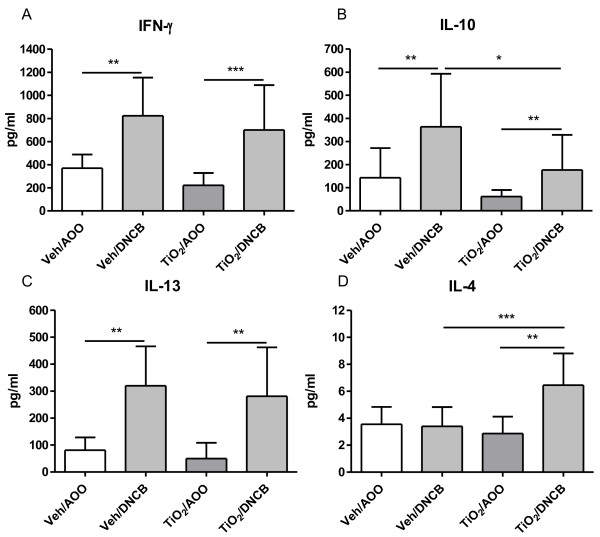
**Lymphocyte cytokine production after 0.1% DNCB dermal sensitization or AOO treatment with prior injection of 0.04 mg/ml TiO2 or vehicle.** On day 5, the auricular lymph nodes are removed and lymphocytes are cultured for 42 h in the presence of ConA. Cytokines are determined in LNC supernatant. Graphs show mean ± SD, n = 10–12, *p < 0.05, ** *p* < 0.01 and *** *p* < 0.001.

Figure 4 shows the levels of cytokine production in cultured auricular lymphocytes, in the presence of ConA. Mice subcutaneously injected with vehicle, followed by 0.1% DNCB sensitization showed a significant increase in the level of IFN-γ (Figure 4A), IL-10 (Figure 4B) and IL-13 (Figure 4C) compared to Veh/AOO group. When the mice were injected with 0.04 mg/ml TiO_2_ NPs before DNCB sensitization, the level of IL-4 (Figure 4D) was also significantly increased, while the level of IL-10 was decreased, but still significantly higher than non-sensitized TiO_2_ NP treated mice. The levels of IFN-γ and IL-13 were unchanged by TiO_2_ NP injection before DNCB sensitization. IL-2 and IL-17 levels were in all groups the same (data not shown).

## Discussion

Our objective was to evaluate the effects of non-biodegradable/biopersistant solid TiO_2_ NPs on the classical hypersensitivity reaction to a well-known potent dermal sensitizer (DNCB). To the best of our knowledge, there is no information available on the effect of manufactured NPs on the dermal sensitization potential of chemicals, in general, and of DNCB in particular. Recently, sensitizing potentials of biodegradable particles (ethosomes) was described, but such information about environmentally relevant solid NPs is lacking [19-21]. Here we demonstrate that TiO_2_ NPs (0.04 mg/ml) act as an immune-stimulator on the dermal sensitization capacity of DNCB. The stimulation of the dermal sensitization was coupled with a significant change in cytokine release which corresponds with the induction of a Th2 response. The experiments confirmed our hypothesis that pre-treatment with TiO_2_ NPs modulate sensitization to DNCB.

There is conflicting evidence about the skin penetration of nanomaterials [22,23]. However, it has been suggested that NPs could pass through the *stratum corneum* of the skin using intercellular channels or hair follicles and penetrate into deeper skin layers [24,25]. A thorough search of the available literature indicates that stratum corneum is an effective barrier against the uptake of TiO_2_ NPs in healthy skin. However, various research publications anticipate the possibility of penetration of TiO_2_ NPs to the deeper layers of skin/viable skin in case of local damage (sun burns etc) or when lipid vesicles formulations are involved [19,20,26]. For example, a sub-erythemal dose ultraviolet radiation exposure (UVB 270 mJ/cm^2^) in mice has been shown to allow the penetration of 45 nm quantum dot NPs to deep dermis [27]. In another study, it has been observed that 7 nm TiO_2_ NP exposure with UV irradiation can lead to increased skin barrier dysfunction and possible aggravation of contact dermatitis due to increased invasion of *Staphylococcus aureus*[28]. Interestingly, people tend to apply more sun screens containing NPs in case they have damaged skin (sunburns, burns) when the barrier function of skin is already impaired. It has also been reported that in a skin barrier dysfunction conditions (mite allergen exposure) intradermal exposure to rutile TiO_2_ NPs can lead to aggravation of the atopic skin lesions [29]. Particles which penetrate normal or damaged skin are taken up by antigen presenting cells (APC), such as Langerhans cells or dendritic cells, and subsequently removed via the lymphatic system [9,30]. In view of these considerations, along with the possibility of exposure in individuals with pathological lesions of the skin, subcutaneous route becomes a relevant route to study NP induced effects on dermal sensitization. TiO_2_ NP concentrations used in this study are based on literature reports by us and others describing these as non-cytotoxic concentrations *in vitro* and *in vivo*[31,32]. Moreover, we did not observe any type of local injury around the site of injection of NPs.

Our results indicate that TiO_2_ NPs do not show dermal sensitization potential after a single subcutaneous injection, which is in accordance with findings in literature [32]. However, subcutaneous presence of TiO_2_ NPs significantly increases the skin sensitization potential of DNCB, which is not the case with subcutaneous presence of pigment TiO_2_ particles before dermal sensitization. It is possible that increased sensitization after NP exposures is due to binding of the antigen with the NPs, leading to formation of depot of antigen which is better recognized by skin APCs. It has been proposed that ultrafine TiO_2_ particles (below 30 nm) bind more ovalbumin per mass unit than fine particles and this binding may lead to a depot of antigen leading to increased antigenicity [33]. Moreover, it is a well accepted fact that professional APCs are more readily stimulated by the particulate antigens, thus adsorption of antigen *per se* can increase the antigenicity [34]. Other possible explanation might be physical interactions between the NPs and APCs. Impurities cannot be a confounding factor in our experiments as there were no detectable amounts present in TiO_2_ NPs utilized in this study.

The results of the present study demonstrate that pre-exposure to TiO_2_ NPs does not interfere with the immune system if followed by a sham dermal treatment (TiO_2_/Veh). However, when pre-exposure to TiO_2_ NPs is followed by DNCB sensitization, a Th2 favoured immune response in regional lymph node cells develops, with increased IL-4 and decreased IL-10 levels, while DNCB itself is a known potent Th1 responder [35]. The apparent IFN-γ secretion confirms the DNCB-induced Th1 response, even with prior injection of TiO_2_ NPs. Nevertheless, TiO_2_ NPs injection followed by DNCB sensitization results in significantly increased levels of IL-4, demonstrating Th2 stimulation. In addition, we found a decrease in IL-10 secretion. IL-10 is a cytokine released by several cell types, such as monocytes, activated T cells, Th2 cells, mast cells and regulatory T cells. IL-10 is capable of inhibiting pro-inflammatory responses and is suggested to play a major role in maintenance of self-tolerance [36]. We think that sub-cutaneous exposure to TiO_2_ in DNCB sensitized mice, decreased IL-10, and thereby allowing the development of a Th2 response, independent of the presence of the Th1 response (levels of IFN-γ are maintained).

Allergic sensitization reactions are the first step against the “foreign” materials, and are either Th1 or Th2 polarized. It has been shown that particles themselves can act as modulating agents in skewing the Th responses. Impact of particles on the skewing of Th response is largely dependent on the chemical nature and characteristics of the materials. Larsen *et al*, found that TiO_2_ NPs promote allergic sensitization to ovalbumin (IgE and IgG1 levels) and thus primes a Th2 dominated immune response [33]. Diesel exhaust can promote both Th1 and Th2 responses [37,38]. Carbon nanotubes either amplify Th1 (MWCNT) or Th2 (SWCNT) or both (MWCNT) responses [39,40]. These studies are done in the models of respiratory allergy using ovalbumin as sensitizing agent. However, respiratory allergic responses have already been shown to be more prone to Th2 skewing while skin sensitization responses are mostly Th1 dependent [41,42]. It is interesting to note that although we observe a skewing of immune response towards Th2 we still observe a shift in the potency of DNCB, with almost a 3-fold fold decrease in EC_3_.

## Conclusion

In conclusion, we have demonstrated that TiO_2_ NPs are effective in modulating the chemical-induced *in vivo* dermal sensitization. They probably act as adjuvant to increase the dermal sensitization capacity of a model chemical skin sensitizer (DNCB). These findings will be helpful in understanding the NPs induced health effects and will help in understanding the interactions of NPs with other sensitizing agents.

## Methods

### Reagents

2,4-Dinitrochorobenzene was purchased from Sigma-Aldrich (Bornem, Belgium). Trichloroacetic acid (TCA) and acetone were obtained from Sigma-Aldrich (Bornem, Belgium). Pentobarbital (Nembutal) was obtained from Sanofi Santé Animale (CEVA, Brussels, Belgium). Acetone-olive oil vehicle (AOO) used to dissolve DNCB was composed of a mixture of 4 volumes of acetone and 1 volume of olive oil (Selection de Almazara, Carbonell, Madrid, Spain). DNCB concentration is given as percent (w/v) in AOO. Hanks Balanced Salt solution (HBSS) was purchased from Invitrogen (Merelbeke, Belgium) and [Methyl-3 H]-thymidine (^3^HTdR) was purchased from ICN Pharmaceuticals (Asse, Belgium).

### Nanoparticles

TiO_2_ NPs (99.9% anatase) of 10–25 nm diameter (15 nm average diameter) were purchased from Sigma-Aldrich (Saint Quentin Fallavier, France). These particles were prepared by sol–gel process and were used without any post production surface treatments/modifications. Pigment TiO_2_ was purchased from Cinkarna (Celje, Slovenia). Freshly prepared NPs suspensions at desired concentrations in 2.5 mM sodium Citrate were utilized to treat the mice.

### Physico-chemical Characterization of NPs

#### *Dynamic Light Scattering*

DLS analysis of TiO_2_ pigment and NPs was performed according to the protocol described by us previously [43]. Briefly TiO_2_ pigment and NPs were diluted in 2.5 mM sodium citrate (pH 6.95, ionic strength (I)515 mM), ultrasonicated and analyzed using Brookhaven 90 Plus instrument (scattering angle: 90°, wavelength: 659 nm, power 15 mW). Correlation functions were analyzed with the Clementine package (maximum entropy method) for Igor Pro 6.02A. This resulted in intensity weighted distribution functions versus decay times. By converting the decay times with instrument parameters and physical parameters to hydrodynamic diameters, an intensity weighted size distribution was obtained. A lognormal fit was applied on each population resulting in the average hydrodynamic diameter of the population.

#### *Zeta Potential (ζ ) Measurements*

Detailed protocol of *ζ* potential measurement is published elsewhere [43] was measured with a Brookhaven 90Plus/ZetaPlus instrument applying electrophoretic light scattering. A primary and reference beam (659 nm, 35 mW), modulated optics and a dip-in electrode system were used. The frequency shift of scattered light (relative to the reference beam) from a charged particle moving in an electric field is related to the electrophoretic mobility of the particle. The Smoluchowski limit was used to calculate the zeta potential from the electrophoretic mobility.

#### *Transmission electron microscopy (TEM)*

Suspensions of the TiO_2_ particles were applied on formvar-coated cupper mesh grids. After drying overnight, the NPs were characterized by transmission electron microscopy (TEM) (JEOL JEM-1200 EX-II, Tokyo, Japan) at a magnification of 20.000-200.000 x.

### Mice

Male BALB/c mice (approximately 20 g, 6 weeks old) were obtained from Harlan (Horst, The Netherlands). The mice were housed in a conventional animal house with 12-hour dark/light cycles. They were housed in plastic cages with filter tops and received lightly acidified water and pelleted food (Trouw Nutrition, Gent, Belgium) *ad libitum*. All experimental procedures were approved by the local Ethical Committee for Animal Experiments.

### Lymph Node Proliferation Assay (LNPA)

LNPA was performed according to the method described previously [17,18]. Briefly, one hour prior to dermal sensitization, on day 0, mice (5–9 animals per group) were injected sub-cutaneously with NP suspension or vehicle (Veh) (2.5 mM sodium Citrate) on the area medial to the implantation of each ear lobe. Subsequently, on days 0, 1 and 2, the mice were given dermal applications (25 μl on each ear) of DNCB in AOO, or AOO alone. On day 5, the mice were injected intravenously in a tail vein with 250 μl of 20 μCi ^3^HTdR solution in HBSS (pH 7.2). Five hours later, the mice were sacrificed by an overdose of Nembutal (90 mg/kg) and auricular lymph nodes were removed, pooled and weighed. A single cell suspension of lymph node cells (LNC) was prepared and LNC were washed two times with HBSS. Subsequently, the LNC were dissolved in 5% TCA and kept overnight at 4°C. ^3^HTdR incorporation was evaluated by β-scintillation counting (Beckman LS 5000CE) and was expressed as disintegrations per minute (dpm). The stimulation index (SI) was calculated as a ratio of ^3^HTdR incorporation in lymphocytes from DNBC-treated mice and AOO-treated controls. A compound having SI >3 is considered to be a biologically relevant dermal sensitizer.

a) In a first experiment, mice were subcutaneously injected with 2x50μl of a 0.004 (low dose), 0.04 (medium dose) or 0.4 (high dose) mg/ml NP suspension, 0.04 (medium dose) mg/ml pigment particles or vehicle on the area medial to the implantation of ear lobes. One hour later, the mice were dermally treated on both ears with (25 μl) of 0.1% DNCB in AOO, or AOO alone and this for 3 consecutive days. On day 5, the LNPA was performed and the SI calculated.

b) In a second experiment, mice were subcutaneously injected with 2x50μl of 0.04 (medium dose) mg/ml NP suspension or vehicle on the area medial to the implantation of ear lobes. Afterwards, the mice were treated on each ear with DNCB (0.025, 0.050 or 0.1%) in AOO, or AOO alone, for 3 consecutive days. On day 5, the LNPA was performed, the SI calculated, from which the EC_3_ (effective concentration yielding a Si of 3) was determined.

### Lymph node cell analysis

In a separate group of animals, mice received an NP injection with 0.04 mg/ml TiO_2_ or vehicle and were afterwards dermally treated for three consecutive days with 0.1% DNCB sensitization in AOO, or AOO alone, but on day 5 no ^3^HTdR was injected and lymph node cells were isolated. The lymph nodes were kept on ice in RPMI-1640 (Invitrogen, Merelbeke, Belgium) and cell suspensions were obtained by pressing the lymph nodes through a cell strainer (100 μm) (BD Bioscience, Erembodegem, Belgium) and rinsing with 10 ml RPMI-1640. Cells were counted using a Bürker hemocytometer. Lymphocytes were washed three times and suspended (10^7^ cells/ml) in complete tissue culture medium (RPMI-1640 supplemented with 10% heat-inactivated fetal bovine serum, 10 mg/ml streptomycin and 100 IU/ml penicillin).

a) Cell subpopulation analysis:

We stained 5x10^5^ cells with either anti-CD3+ (APC, T-lymphocytes), anti-CD4+ (APC-Cy7, Th-lymphocytes), anti-CD8+ (PerCP-Cy5.5, Tc-lymphocytes) and anti-CD25+ (PE, activated/regulatory T-lymphocytes) antibodies or with anti-CD19+ (PE, B-lymphocytes) labelled antibodies, according to manufacturer’s instructions (BD Biosciences, Erembodegem, Belgium). All necessary controls (including isotype controls) were performed using isotype match control antibodies. Flow cytometry (FACSArray, BD Biosciences, Erembodegem, Belgium) was performed using at least 10^5^ cells.

b) Cytokine analysis:

Cells were seeded into 48-well culture plates at a density of 106 cells/ml and incubated in complete RPMI-1640 medium for 42 h containing 2.5 μg/ml concanavaline A (ConA) (Sigma–Aldrich, Bornem, Belgium). Culture supernatant was collected, centrifuged (1000 g, 15 minutes at 4°C) and stored at −80°C till further analysis. Levels of IL-2, IL-4, IL-10, IL-13, IL-17 and interferon gamma (IFN-γ) were measured via Cytometric Bead Array and analyzed with the FCAP Array Software on the FACSArray (BD Biosciences, Erembodegem, Belgium). Lower detection limits were 0.2 pg/ml, 0.3 pg/ml, 9.6 pg/ml, 2.4 pg/ml, 0.95 pg/ml and 0.5 pg/ml, respectively.

### Statistical Analysis

Data are presented as means and SD. All the data were log transformed for statistical analysis (Graphpad Prism 5.01, Graphpad Software, Inc, San Diego, CA). Data of Table 1, Figures 2 and 4 were analysed by a one-way ANOVA followed by Bonferroni post hoc test. In the post-hoc test, four different comparisons were performed: Veh/AOO vs. Veh/DNCB, Veh/AOO vs. TiO_2_/AOO, TiO_2_/AOO vs. TiO_2_/DNCB and Veh/DNCB vs. TiO_2_/DNCB. Figure 3 was analysed using a two-way ANOVA followed by a bonferroni post hoc test, comparing Veh vs TiO_2_ injection prior to DNCB sensitization. A level of *p* < 0.05 (two tailed) was considered as significant.

## Competing interests

None of the authors have competing interests.

## Authors’ contributions

SH and SS were involved in setting up and performing the experiments, along with writing the manuscript. VDV and BE performed the experiments and read the manuscript. KVL performed TEM imaging. SB and FM are the promoter of SH and thoroughly read the manuscript. BN, PH and JV are the promoters of SS, BE and VDV. They came up with the idea for the experiment, initiated the set-up, read the manuscript and are responsible for its final version.
